# National Aquatic Resource Surveys (NARS): the foundation for long-term aquatic monitoring data across the United States

**DOI:** 10.1007/s10661-025-14629-8

**Published:** 2025-11-03

**Authors:** Amanda M. Nahlik, Steven G. Paulsen, Michael Dumelle, Susan Holdsworth, Sarah Lehmann, Nicolle S. Tulve, Sean J. Paul, H. Christopher Frey

**Affiliations:** 1 United States Environmental Protection Agency, Office of Research and Development, Center for Public Health and Environmental Assessment, Pacific Ecological Systems Division, Corvallis, OR USA; 2 United States Environmental Protection Agency, Office of Water, Office of Wetlands, Oceans and Watersheds, Washington, D.C USA; 3 United States Environmental Protection Agency, Office of Research and Development, Center for Public Health and Environmental Assessment, Public Health and Environmental Systems Division, Research Triangle Park, NC USA; 4 United States Environmental Protection Agency, Office of Research and Development, Washington, DC USA; 5 United States Environmental Protection Agency, Assistant Administrator for the Office of Research and Development and EPA Science Advisor, Washington, DC USA; 6 Present Address: College of Engineering, North Carolina State University, Raleigh, NC USA

**Keywords:** National monitoring, Metrics and indicators, National Lakes Assessment (NLA), National Rivers and Streams Assessment (NRSA), National Coastal Condition Assessment (NCCA), National Wetland Condition Assessment (NWCA)

## Abstract

There is a common saying in business that “what matters gets measured”. Environmental protection and sustainability certainly should qualify as important concerns for both businesses and governments. The 1970s marked the genesis of the modern environmental movement, with growing concern about water pollution in the United States (U.S.). In 1972, the U.S. enacted the Clean Water Act (CWA) “*to restore and maintain the chemical, physical, and biological integrity of the Nation’s waters*”. In accordance with CWA objectives, the U.S. Environmental Protection Agency (EPA) implemented the surface water and estuary regional research components as part of the Environmental Monitoring and Assessment Program (EMAP), from which the National Aquatic Resource Surveys (NARS) evolved. NARS is designed to assess the condition of all the Nation’s aquatic ecosystems, including lakes and reservoirs, rivers and streams, coastal waters, the Great Lakes, and wetlands. NARS is the only long-term program that, in collaboration with states and Tribes, monitors the chemical, physical, and biological integrity of the Nation’s aquatic ecosystems using a statistical survey design and consistent field protocols. In this article, we discuss why NARS exists, its key objectives, how NARS has influenced monitoring of natural resources, and how NARS data are used to support U.S. research and policy decisions at broad spatial and temporal extents. We also provide specific examples of how NARS data support condition and risk reporting of indicators, including benthic macroinvertebrates and water surface total phosphorus, and development of tools for characterizing emerging concerns, such as harmful algal blooms (HABs) and antibiotic resistance genes (ARGs).

**Supplementary Information:**

The online version contains supplementary material available at 10.1007/s10661-025-14629-8.

## A brief history of water quality monitoring in the U.S.

The United States (U.S.) relies on water for transport, food and drinking water, recreation, and countless other services (Allan & Flecker, 1993; Covich, 1993; Peterson & Lubchenco, 1997; Postel & Carpenter, 1997). With the rise of industrial America in the late 19th and early twentieth centuries, the U.S. began to exploit their aquatic resources (Strayer & Dudgeon, 2010). Rivers and streams were channelized, diverted, and dammed; wetlands were drained for agriculture and housing; and industrial and municipal wastes were dumped into waterbodies to “dilute” or transport the pollution elsewhere. As a result, many of the waters failed to support fish, shellfish, and other organisms; some of the waters were too toxic to drink or to use for recreation. U.S. waters threatened human health and biodiversity.

Mounting public outrage over the state of the environment gave rise to the modern U.S. environmental movement in 1970. The ecological disasters associated with the fire on Ohio’s Cuyahoga River and California’s Santa Barbara oil spill served as a call to action, culminating in the first Earth Day on April 22, 1970 (Conglianese, 2001). Congress responded by establishing the U.S. Environmental Protection Agency (EPA) in 1970 and enacting the Clean Water Act (CWA) in 1972 under EPA’s directive. Within the CWA is the charge for measuring and reporting on the Nation’s progress in meeting the Act’s objectives – “*to restore and maintain the chemical, physical, and biological integrity of the Nation’s waters*” (CWA§101(a)). The CWA reflects a shared responsibility for monitoring our waters to meet the various needs for water body information. A substantial portion of the responsibility is delegated to the states, but §305(b) directs EPA and states to report on the condition of the Nation’s waters and §104(a)(5) requires EPA to work with partners to establish a national program to monitor water quality. Whereas the states monitor waters of statewide importance, each state functions independently, implementing its own survey and site-scale designs, using different sampling methods, measuring different indicators, and setting different criteria for acceptable water body condition. The resulting data may be valuable at the state level, but they cannot be combined to comprehensively and rigorously assess the national progress in meeting CWA objectives. Several seemingly simple questions could not be answered, such as: What is the ecological condition (status) of U.S. waters? Is the condition improving or worsening (trend)? How widespread are problems (extent)? What are the primary causes of problems (risks)? Are regulatory policies effective or should our energy and financial resources be focused elsewhere (actions)? Such questions could not be answered in part because of the lack of standardization amongst state monitoring approaches that result in disparate, incompatible data. Simply put, EPA faced challenges meeting the national charge of the CWA objective (NRC, 1977; GAO, 1981; GAO, 1988; NRC, 1996; GAO, 2000; The Heinz Center, 2002).

Beginning in the 1990 s, EPA’s Office of Research and Development (ORD) began organizing and conducting research with the goal of establishing the methods to support a national aquatic resource monitoring program. The regional-scale research under the surface water component of Environmental Monitoring and Assessment Program (EMAP) (Messer et al., 1991; Whittier & Paulsen, 1992) was supported by EPA for over two decades and established four key monitoring components. 1) Reliable and repeatable field and laboratory methods were developed to collect data from aquatic resources across the country regardless of variations within the sampled water body (Lazorchak et al., 2000). 2) Statistical (i.e., probability-based) survey design techniques were developed to produce unbiased estimates of condition that support inferences to a broader population of aquatic resources with documented confidence intervals (Diaz-Ramos et al., 1996; Herlihy et al., 2000; Olsen et al., 1999; Stevens & Olsen, 1999, 2003, 2004). 3) Consistent sets of physical, chemical, and biological indicators were determined for each water body type. 4) Appropriate analyses were produced to provide comprehensive assessments of the chemical, physical, and biological condition of the Nation’s waters (e.g., Hughes et al., 2000; Larsen et al., 1991). These monitoring components established under EMAP provided a crucial foundation for the development of the National Aquatic Resource Surveys (NARS).

## National Aquatic Resource Surveys (NARS)

NARS assesses progress towards CWA objectives and is the only program in the U.S. that collects field-based data on the status and trends of aquatic ecosystem conditions at both regional and national extents using a statistical survey design. NARS is a collaboration between EPA’s ORD and Office of Water (OW) and is conducted in partnership with the ten EPA Regional Offices, the states, and Tribes. Surveys are conducted across the conterminous U.S. annually, with 1-day field visits by participating states, Tribes, EPA Regions, universities, and contractors. Co-located chemical, physical, and biological samples and data are collected at each of approximately 1,000 sites by using standardized field and laboratory protocols that ensure both field crews and laboratories are consistent with each other, and across different survey cycles.

**Fig. 1:**
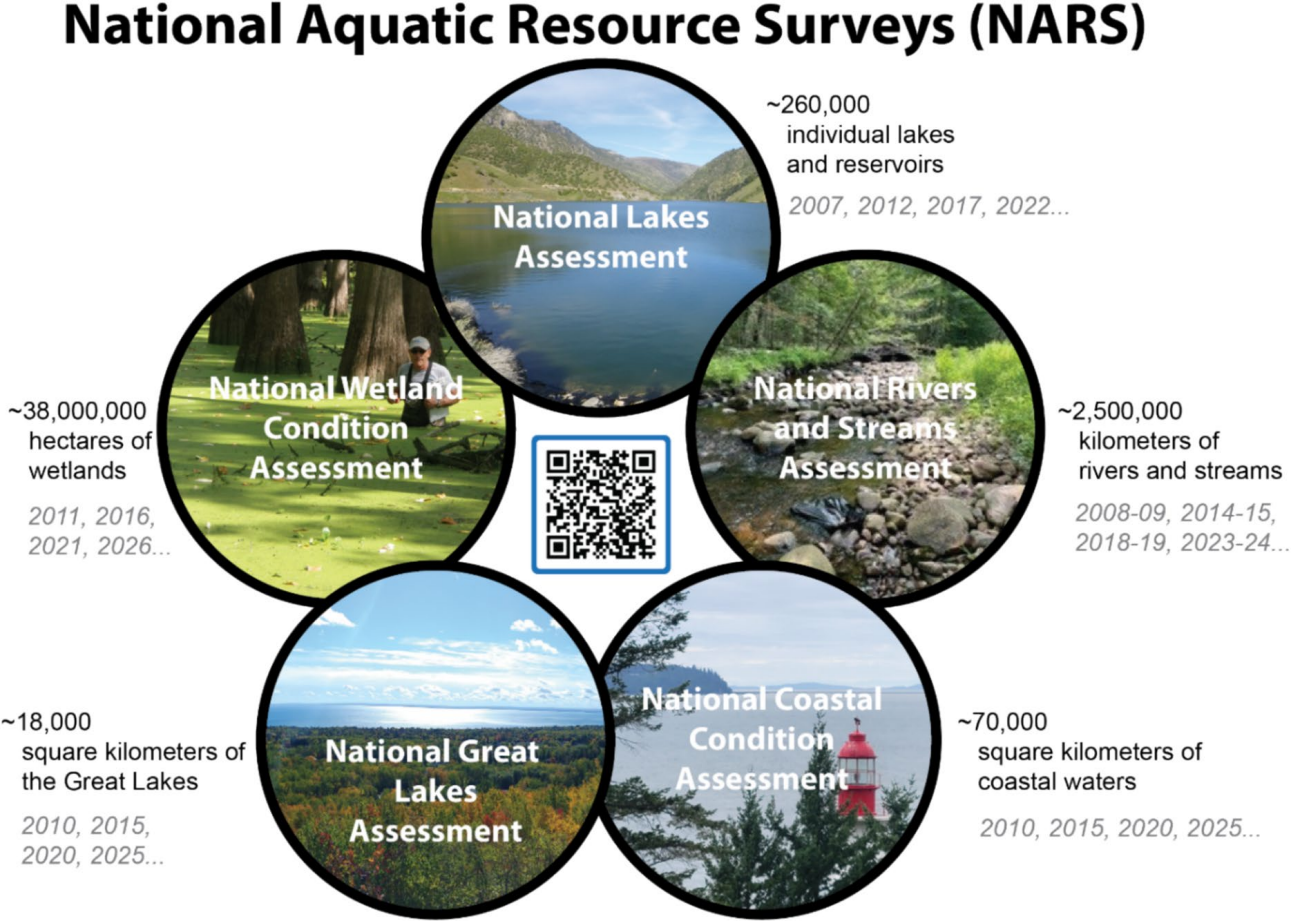
Five assessments included in the National Aquatic Resource Surveys (NARS). The target population, or the area or length (extent) of the aquatic resource across the conterminous U.S. for which national estimates are made, is detailed next to each survey. Years in which each survey was conducted or is planned are italicized in gray text. The QR code may be scanned to reach the U.S. EPA NARS website (https://www.epa.gov/national-aquatic-resource-surveys), where reports, data dashboards, publications, and data are available (see also Supplementary Table 1)

Aquatic resources are surveyed annually on a 5-year cycle. Lakes and reservoirs (National Lakes Assessment (NLA)) are sampled in year 1. Rivers and streams (National Rivers and Streams Assessment (NRSA)) are sampled in years 2–3 because of their extent. Estuarine coastal waters and nearshore Great Lake waters (National Coastal Condition Assessment (NCCA) and National Great Lakes Assessment (NGLA)) are sampled in year 4. And wetlands (National Wetland Condition Assessment (NWCA)) are sampled in year 5. Thus, a full cycle of all aquatic resources is conducted every five years before being repeated (Fig. 1). NARS, which meets criteria under CWA§104(a) and §305(b), is expected to be conducted indefinitely, producing characterizations of condition and long-term trends.

One of the many revolutionary and integral aspects of NARS is the statistical survey design by which field sites are randomly selected and spatially balanced (Peck et al., 2013; Olsen et al., 2012, 2019). The statistical survey design enables valid statistical inferences to be made for parameters, or quantities of scientific interest requiring estimation, e.g., the true proportion of lakes across the conterminous U.S. in good condition for total phosphorus in water. Hereon, we use the term “population estimates” to mean estimates of parameters in the target population, or U.S. waters that meet a target definition (Table 1). Outside of NARS, most ecological field work is conducted at judgmental sites (i.e., non-random) because of ease of access or a particular concern. But research based on judgmental sites and scaled to larger areal units (i.e., county, state, region, Nation), often results in biased, unrepresentative conclusions (Hughes et al., 2000; Paulsen et al., 1998). Because of the statistical survey design that ORD developed under EMAP and that NARS further popularized, many programs and studies – at extents ranging from watersheds to states to countries – have now adopted this survey approach. Applications range from methane emission rates in an Ohio reservoir (Beaulieu et al., 2016), wetland condition in a Minnesota watershed (Genet & Olsen, 2008), Maryland statewide stream condition (Southerland et al., 2009), fish contaminants in U.S. lakes (Stahl et al., 2009), and U.S. stream and lake ecological conditions (Olsen & Peck, 2008; Peck et al., 2013; 2009). The same survey design, site-sampling methodologies, and similar ecological indicators were applied for assessing streams in large Brazilian hydrologic units (Jiménez-Valencia et al., 2014; Silva et al., 2018; Martins et al., 2021; Amaral et al., 2025) and fish assemblage patterns in large, complex Brazilian reservoirs (Becker et al., 2016; Sanches et al., 2016). A similar probability survey design with consistent, quantitative indicator sampling methods has also been implemented in Australia’s Murray-Darling River basin (Davies et al., 2010). Additionally, several other national-scale programs within the United States employ probabilistic monitoring of resources other than water, such as the U.S. Forest Service Forest Inventory and Analysis (FIA; Smith, 2002) and U.S. Geological Survey North American Bat Monitoring Program (NABat; Loeb et al., 2015). More broadly, statistical survey designs are regularly used for election polling, human health statistics, agricultural commodity production, and many other applications.

**Table 1:** Target population definitions for each survey, including National Lakes Assessment (NLA), National Rivers and Streams Assessment (NRSA), National Coastal Condition Assessment (NCCA), National Great Lakes Assessment (NGLA), and National Wetland Condition Assessment (NWCA)

NLA	NRSA	NCCA	NGLA	NWCA
The target population of “lakes” includes natural and man-made freshwater lakes, ponds, and reservoirs: • greater than one hectare (approximately 2.5 acres), • greater than 1,000 square meters of open water, • greater than one meter in depth, • non-saline due to saltwater intrusion or tidal influence, and • not used for aquaculture, disposal-tailings, mine-tailings, sewage treatment, evaporation or other unspecified disposal use that are within the conterminous U.S., excluding the Great Lakes. *Reference: U.S. EPA (2017). National Lakes Assessment 2017. Field Operations Manual. U.S. EPA, Office of Water, Washington, DC. EPA 841-B-16–002*	The target population consists of all streams and rivers within the 48 contiguous states that have flowing water during the study index period, including major rivers and small streams. Sites must have > 50% of the reach length with standing water, and sites with water in less than 50% of the reach length must be dropped. All sites must be sampled during base flow conditions. The target population excludes: • Tidal rivers and streams up to head of salt (defined as < 0.5 ppt for this study); and, • Run-of-the-river ponds and reservoirs with greater than seven-day residence time. *Reference: U.S. EPA (2017). National Rivers and Streams Assessment 2018/19. Field Operations Manual – Wadeable. U.S. EPA Office of Water, Washington, DC. EPA 841-B-17-003a*	The target population for the estuarine resources consists of all coastal waters of the conterminous United States from the head-of-salt to confluence with the ocean, including inland waterways, tidal rivers and creeks, lagoons, fjords, bays, and major embayments. For the purposes of this study, the head-of- salt is defined as waters with salinity less than 0.5 practical salinity units (psu) salinity, representing the landward/upstream boundary. The seaward boundary extends out to where an imaginary straight-line intersecting two land features would fully enclose a body of coastal water. All waters within the enclosed area are defined as estuarine, regardless of depth or salinity. *Reference: U.S. EPA (2020). National Coastal Condition Assessment. Field Operations Manual. U.S. EPA, Office of Water, Washington, DC. EPA 841-F-19–005*	The target population for the Great Lakes consists of all waters of the Great Lakes of the United States and Canada. The current target population is restricted to the shallow nearshore zones of Lake Superior, Lake Michigan, Lake Huron, Lake Erie, and Lake Ontario. The NGLA Great Lakes sites are restricted to waters within the United States. *Reference: U.S. EPA (2020). National Coastal Condition Assessment. Field Operations Manual. U.S. EPA, Office of Water, Washington, DC. EPA 841-F-19–005*	The target population for NWCA is tidal and nontidal wetlands of the conterminous U.S., including certain farmed wetlands not currently in crop production. The wetlands have rooted vegetation and, when present, open water less than 1 m deep. Note: The NWCA defines wetlands using the classification system described by Cowardin et al. (1979)^1^ and established as a Federal Geographic Data Committee (FGDC) standard for classification of wetlands. This may be different than the definitions applied under state or federal regulatory programs. A wetland’s status under state or federal regulatory programs does not affect a site’s status as target for purposes of NWCA. *Reference: U.S. EPA (2021). National Wetland Condition Assessment. Field Operations Manual. U.S. EPA, Office of Water, Washington, DC. EPA 843-R-10–001*

^1^ Cowardin, L.M., Carter, V., Golet, F.C., & LaRoe E.T. (1979). *Classification of wetlands and deepwater habitats of the United States*. U.S. Department of the Interior, Fish and Wildlife Service, Washington, D.C

As of 2024, three full NARS cycles have been completed, sampling 3,071 lakes and reservoirs, 5,628 river and stream locations, 2,196 coastal water and 984 Great Lake sites, and 2,867 wetland sites. Leveraging the survey design, these data are used to estimate target population parameters for all of the conterminous U.S. (Fig. 1). The field and laboratory data, along with the survey design information (using the R package *spsurvey*; Dumelle et al., 2023), are used to estimate the proportion of the resource population (also called "extent", i.e., units (e.g., lakes), kilometers, square miles, or hectares) in Good, Fair, and Poor ecological condition for each resource type across the conterminous U.S. with documented confidence (i.e., confidence intervals). Condition thresholds used to determine Good, Fair, and Poor categories vary by indicator type (e.g., multimetric indices (MMIs), analytes, metrics) and can vary by scale (national or regional) and by ecosystem characteristics (e.g., wetland types, wadeable versus boatable rivers and streams). In most cases, it is important to consider regional and physical differences in condition thresholds because human impacts to aquatic ecosystems have occurred to varying degrees across the country. Likewise, “Good” condition typically represents the best available condition, as all ecosystems have been directly or indirectly impacted by human activities (Stoddard et al., 2006; Vitousek, 1994).

**Fig. 2:**
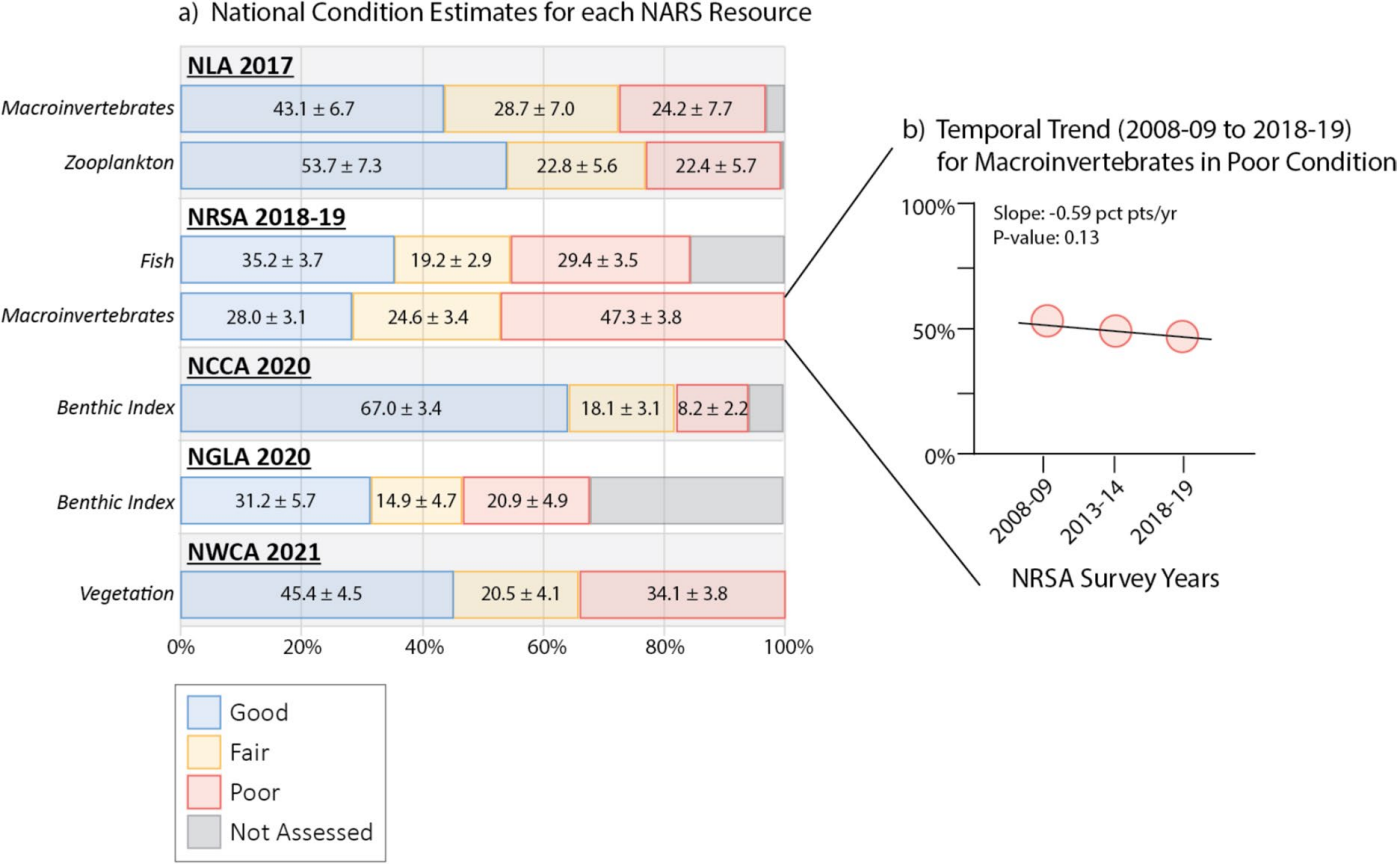
**a**) National condition estimates characterizing the proportion of Good, Fair, Poor, and Not Assessed ecological condition for each NARS resource during Cycle 3 (2017–2021). Biological indicators for each survey are reported on the y-axis in italics. Condition estimates are reported for each bar in percent ± 95% margin of error. “Not Assessed” condition represents the proportion of the target population that was not measurable because of e.g., site conditions (e.g., hard bottom), field crew safety, absence of at-risk species permits (e.g., salmonids), absence of the indicator, etc. **b**) Temporal trend for poor ecological condition as indicated by macroinvertebrates measured in NRSA from 2008–09 to 2018–19. Individual survey (point) estimates are 53.2, 49.3, and 47.3% for 2008–09, 2013–14, and 2018–19, respectively. Supplementary Table 1 provides the URLs for survey results and details

The most recently completed NARS Cycle (Cycle 3; 2017–2021) for many aquatic resources shows that the proportion of those resources in Fair and Poor ecological condition is greater than that in Good condition (Fig. 2a, United States Environmental Protection Agency, 2022, 2023, 2024a, 2024b.). This is important information about the state of our aquatic resources and the ecosystem services depending on them. Perhaps even more importantly, the cyclic nature of NARS allows us to track temporal trends in ecosystem conditions to assess whether the Nation is meeting the CWA objectives of maintaining or improving aquatic resource condition. Figure 2b provides an example of how trends may be estimated using the proportion of macroinvertebrates in poor condition from NRSA 2008–09, 2013–14, and 2018–19. In this case, the national proportion of rivers and streams in poor condition reflected by macroinvertebrates has remained relatively stable (p = 0.13) across the three surveys. Continuation of the surveys allows for trends to be more strongly detected (i.e., statistically significant) in the future.

In another example that has shed light on the understanding of ecological condition of the nation’s aquatic resources, Stoddard et al. (2016) examined long-term concentrations of surface water total phosphorus (TP) in lakes (2007 and 2012 NLA) and rivers & streams (2000–2004 EMAP, 2008–2009 and 2013–2014 NRSA). They found continental-scale increases in TP concentrations in both lakes and rivers & streams, with the greatest change over time in naturally oligotrophic systems (i.e., < 10 µg TP L^−1^). The authors suggested several potential causes for widespread increases in TP, which has contributed to both national and global research efforts into landscape-scale drivers of TP inputs and mobilization, with the goal of informing resource managers (e.g., Bol et al., 2018; Sabo et al., 2023).

**Fig. 3:**
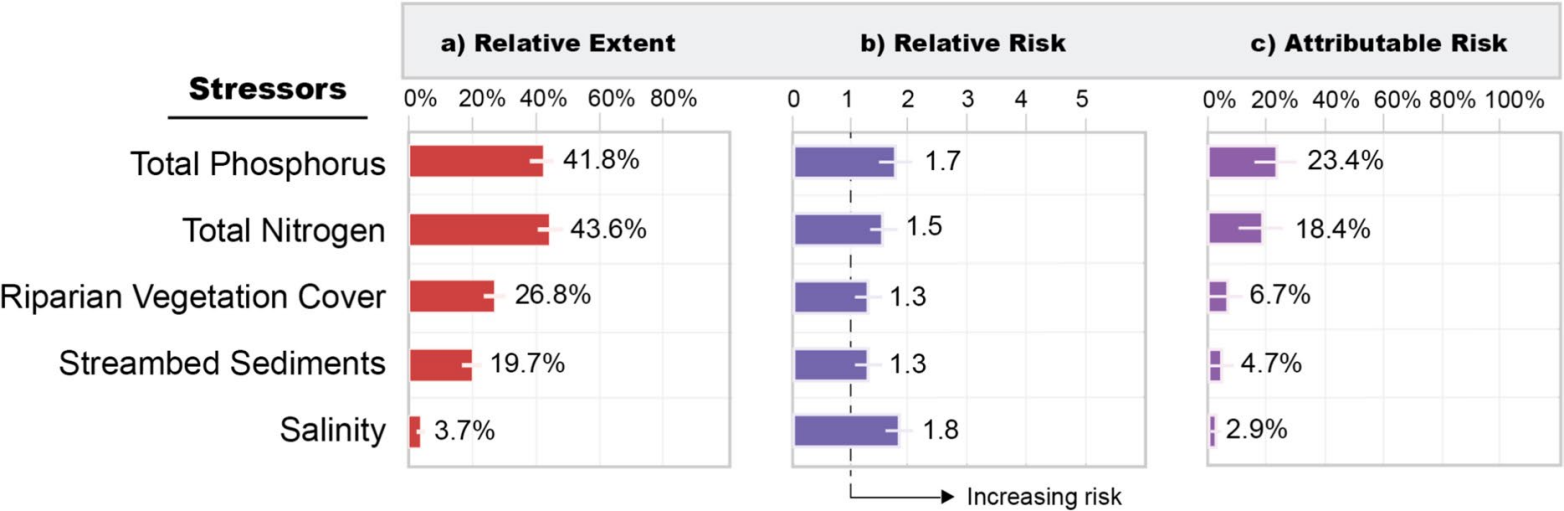
Examples of **a**) relative extent (percent of river and stream miles), **b**) relative risk (unitless), and **c**) attributable risk (percent of river and stream miles) associated with Poor ecological condition as indicated by macroinvertebrates measured in NRSA 2018–19. Note that a relative risk value of ≤ 1 indicates no association between the stressor and ecological condition

In addition to monitoring the status of aquatic resource condition and trends in that condition over time, NARS uses several tools to help inform progress in meeting the CWA goals – relative extent, relative risk, and attributable risk (Fig. 3). In the context of risk, relative extent is defined by NARS as the estimated proportion of an aquatic resource population in Poor stressor condition, which provides information about how widespread a stressor is. NARS data are also used to describe the relative and attributable risk posed to an aquatic resource by a particular stressor, thus giving us actionable information on the major stressors most strongly associated with the problems. Relative risk is defined as the probability (risk or likelihood) of having Poor resource condition when a chemical or physical indicator is in Poor condition relative to when the indicator is in Fair or Good condition (Van Sickle et al., 2006). Attributable risk estimates the proportional reduction in the extent of Poor condition aquatic resource that might be achieved if the stressor were reduced or eliminated (Van Sickle & Paulsen, 2008). The use of relative and attributable risk in the context of NARS is not unlike that of their more traditional application to medical risk (e.g., having a greater relative risk of developing heart disease if one has high cholesterol levels compared to low cholesterol levels). NARS adopted this approach because it is a useful method for estimating associations between two groups, as well as an effective communication tool for explaining results to the public.

We provide an example using the NRSA 2018–19 macroinvertebrate (ecological) condition and its relationship to measured stressors of how relative extent, relative risk, and attributable risk are reported. Figure 3a shows the estimated proportion (i.e., relative extent) of river and stream length in the conterminous U.S. with high stressor levels; for example, 41.8% of river and stream miles had high total phosphorus (TP) levels measured in the water column. When relative risk for TP is calculated (Fig. 3b), we see that rivers and streams with high TP levels are 1.7 times more likely to be in Poor ecological condition than rivers and streams with low TP levels (see U.S. EPA 2022 for documentation on how benchmarks between low, moderate, and high stressor levels were determined). Considering both the relative extent *and* relative risk together, we can estimate the attributable risk, which, in the case of TP (Fig. 3c), shows that 23.4% of the river and stream length in Poor ecological condition could be improved to Good. By ranking the measured stressors using their attributable risk scores, insight can be gained about which stressors resource managers may want to prioritize to improve ecosystems in Poor condition. In our example, if nutrient inputs– total phosphorus and total nitrogen – we reduced or eliminated in rivers and streams across the U.S., a substantial proportion of the Poor condition rivers and streams may be improved.

NARS data are also used to estimate averages, percentiles (e.g., first quartile, median, third quartile), and distribution functions for continuous variables (e.g., perfluorooctane sulfonate (PFOS), total mercury, and polychlorinated biphenyl (PCB) concentrations in fish tissue; Stahl et al., 2023). Resource condition and risk results and trends over time for core indicators (Table 2) are reported by OW in online reports and interactive dashboards (Supplementary Table 1).

**Table 2:** List of condition indicators included in the National Aquatic Resource Survey (NARS) reports produced by EPA, including National Lakes Assessment (NLA), National Rivers and Streams Assessment (NRSA), National Coastal Condition Assessment (NCCA), National Great Lakes Assessment (NGLA), and National Wetland Condition Assessment (NWCA)

	*Chemical*	*Physical Habitat*	*Biological*	*Human Health*
*NLA*	Acidification (ANC) Atrazine Dissolved Oxygen Total Nitrogen Total Phosphorus	Lake Drawdown Exposure Lake Habitat Complexity Lakeshore Disturbance Riparian Vegetation Cover Shallow Water Habitat	Chlorophyll-*a* Benthic Macroinvertebrates Zooplankton	Microcystins Mercury in Fish Tissue
*NRSA*	Acidification (ANC) Total Nitrogen Total Phosphorus Salinity (Conductivity)	In-Stream Fish Habitat Riparian Disturbance Riparian Vegetative Cover Streambed Sediments	Macroinvertebrates Fish	Enterococci Microcystins Mercury in Fish Tissue
*NCCA*	Eutrophication Index * Dissolved Oxygen* * Water Clarity* * Chlorophyll-a* * Nitrogen (dissolved)* * Phosphorous (dissolved)* Total Phosphorus Total Nitrogen Sediment Quality Index * Sediments Contaminants* * Sediment Toxicity*	N/A	Benthic Index	Fish Quality Index Enterococci Microcystins Mercury in Fish Tissue*
*NGLA*	Eutrophication Index * Dissolved Oxygen* * Water Clarity* * Chlorophyll-a* * Total Phosphorus* Total Nitrogen Sediment Quality Index * Sediments Contaminants* * Sediment Toxicity*	N/A	Benthic Index	Fish Quality Index Enterococci Microcystins Mercury in Fish Tissue
*NWCA*	Soil Heavy Metals Total Nitrogen (in water) Total Phosphorus (in water)	Physical Alterations (Sum) Vegetation Removal Vegetation Replacement Addition/Subtraction Flow Obstruction Soil Hardening Surface Modification	Vegetation Multimetric Index Nonnative Plants	Microcystins

Note that this list is not exhaustive of all indicators under development

## NARS as a foundation and source of data

NARS produces arguably the most comprehensive national-extent datasets for aquatic ecosystems in the world. These large datasets, containing field measurements, laboratory analytical results, and condition estimates, are thoroughly verified and validated, documented, and publicly available through the NARS website. NARS data are used as the foundation for answers to unexpected questions, leveraged to inform state, territory, and Tribe water quality criteria and threshold recommendations (e.g., United States Environmental Protection Agency, 2021, 2024c), and support a substantial amount of research across EPA. No less than 10% of the planned research between 2023 and 2026 in ORD (United States Environmental Protection Agency, 2024d) relies on NARS data. Influential research produced by EPA that used NARS data ranges from social science (Hill et al., 2023) to soil carbon storage (Nahlik & Fennessy, 2016). NARS data supports human health risk research, e.g., toxic cyanobacteria, fish fillet tissue contaminant studies, harmful algal blooms, microbial antibiotic resistance genes (Stahl et al., 2009; Wathen et al., 2015; Keeley et al., 2022; Handler et al., 2023; Hughes et al., 2024) and research focused on environmental shifts (Lin et al., 2021; Rumschlag et al., 2023), among others. In addition, the value of NARS data in answering important ecological questions is becoming increasingly recognized outside EPA. This is evidenced by the hundreds of publications produced by scientists, resource managers, and policy makers in state and government agencies, NGOs, and colleges and universities (Supplementary Table 1).

**Fig. 4:**
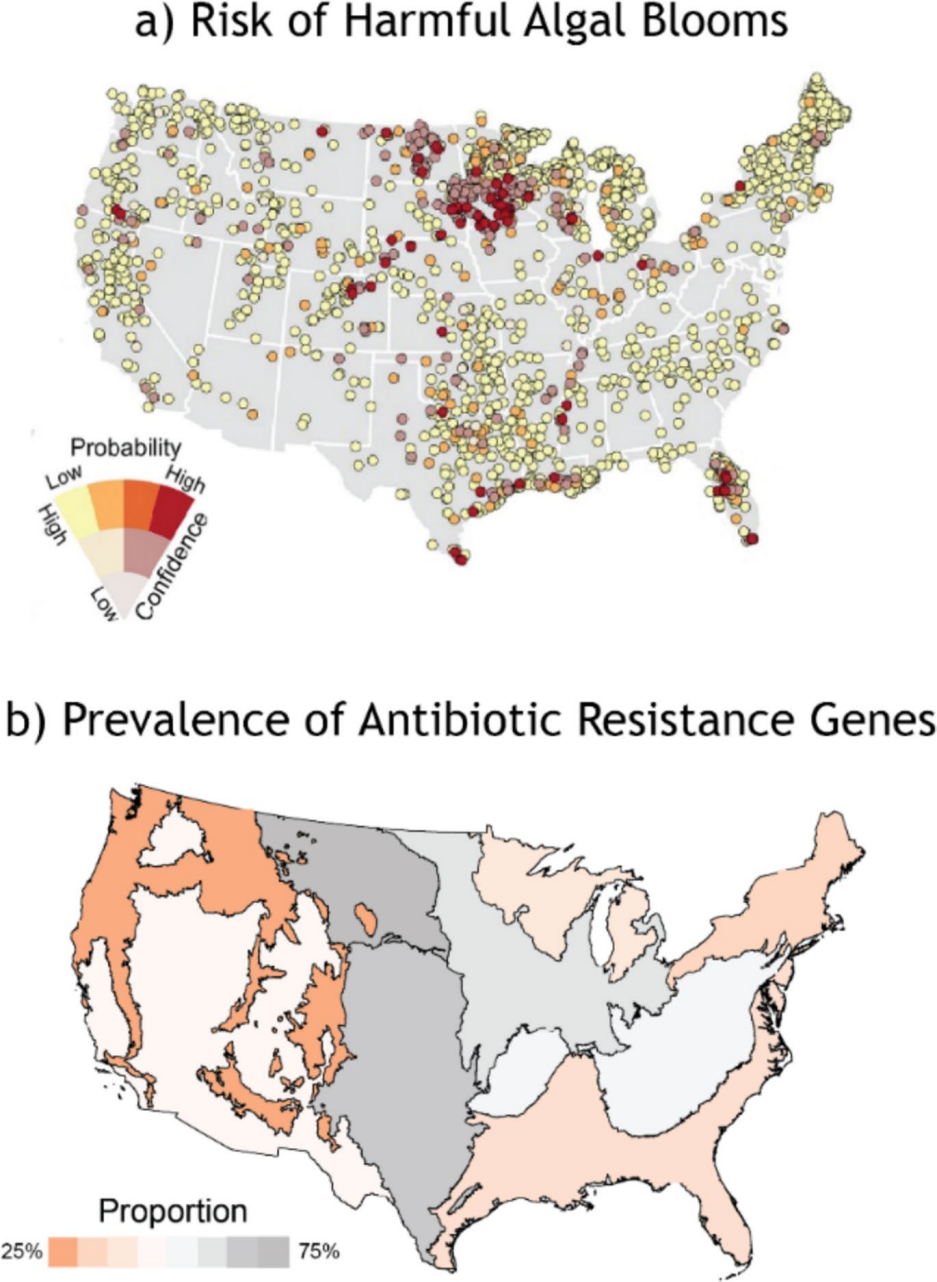
Examples of national-extent research supported by National Aquatic Resource Surveys (NARS) data, including a) risk of harmful algal blooms (HABs) (i.e., microcystin > 0.2 μg/L) in lakes sampled in the National Lake Assessment (NLA) (Handler et al., 2023) and b) prevalence of antibiotic resistance genes (ARGs) (specifically for tetracycline) in streams sampled in the National River and Stream Assessment (NRSA) (Keeley et al., 2022). Points in **a**) are color coded by probability, with low = yellow and high = red, and the saturation of each point reflecting the confidence of the probability estimate, with highly saturated = high and pale = low. Colors in **b**) reflect the estimated proportion of river and stream length within an Ecoregion that exceed reference values of ARGs

NARS data is versatile. Handler et al. (2023) used data from NLA combined with satellite imagery to build a statistical model characterizing the risk of harmful algal blooms (HABs) in lakes across the U.S. The authors found that bloom magnitude was associated with thresholds of microcystin, cyanobacteria, and chlorophyll-*a*. In Fig. 4a, the authors display HABs risk based on a microcystin threshold of > 0.2 μg/L for each lake measured in NLA. In addition to providing useful information about individual lakes, these data elucidate national-scale patterns (Fig. 4a) – both scales of which are important for resource managers to consider. Moreover, the HABs model developed by Handler et al. can be applied to quantify HABs risk at lakes not sampled in the NLA. A separate approach to using NARS data is highlighted by Keeley et al. (2022), who more directly used the statistical survey design to estimate microbial antibiotic resistance genes (ARGs) in rivers and streams across nine ecoregions in the conterminous U.S. Using filtered water samples collected from NRSA, the authors measured targeted genes associated with antibiotic resistance to drugs commonly used to treat human infections. Figure 4b shows the estimated proportion of antibiotic resistance to tetracycline by ecoregion, with, e.g., up to 75% of rivers and streams in the Plains regions (in dark grey) exceeding reference thresholds. In this ARGs example, the authors characterize trends in all targeted rivers and streams across broad regions instead of individual locations in the HABs example.

NARS was envisioned as a program that would produce useful data for evaluating the policies and actions for governing and managing aquatic resources across the country. Based on these assessments, policies ideally would be revised and improved to advance the Nation toward meeting the CWA objectives. NARS was also envisioned as a source of data that could drive resource priorities and provide data for research. The results from the surveys can be used to set priorities within EPA’s ORD and OW and support enhancements in state and Tribal monitoring and assessment programs. An ongoing program such as NARS provides the basis for more quickly answering unforeseen policy questions than our past approaches. For example, in the 1980 s, the U.S. was concerned about the effects of acid rain (Likens et al., 1979), and it took more than 10 years to design a survey, implement it, and produce final condition reports (Baker et al., 1991; Kaufmann et al., 1992). Having a broad, ongoing survey allows the U.S. to bypass the extended process in setting up a problem-specific monitoring program. NARS provides an important source of consistent baseline data not otherwise available, and the surveys can be adapted (e.g., by adding new measurements) to address unanticipated issues as they arise.

NARS has established the foundation for EPA and the U.S. to assess our aquatic resources in the past, present, and future, helping measure our collective progress toward meeting CWA objectives. But NARS and the data produced are also a nexus across research and policy. Data aid EPA Regions, states, Tribes, universities, and others to conduct collaborative research. The NARS Program, which includes staff, laboratory services, and tested field and data analysis methodologies, provides support to states that are mandated to report on the status of their waters under the CWA (e.g., CWA§303(d) and §305(b)). For example, Rehn et al. (2007) conducted a study in northern California streams to compare a widely used riffle (targeted) method of macroinvertebrate sampling to a reach-wide (representative) method of macroinvertebrate sampling developed in EMAP (and continued in NARS). They concluded that the two methods were similar for some stream types, but as the proportion of the reach in the targeted habitat type differs from the representative sample, the condition estimates between the two methods diverged. California ultimately adopted the representative (NARS) approach for their state water monitoring programs. In New Mexico, Jessup et al. (2014) developed a system for deriving numeric benchmarks of fine and unstable sediments in perennial streams that could be used by state environmental agencies to translate their narrative sediment standards into actionable criteria thresholds under CWA§305(b). The authors' recommended benchmarks and supporting analyses provide a strong basis for New Mexico Environment Department to make final selections of sediment thresholds and to establish procedures for their application and ultimately to protect their state’s stream ecosystems (New Mexico Environment Department, 2025). While components of NARS can support local and state monitoring programs and their progress towards meeting CWA goals, the national scope of NARS data primarily informs us about the aggregate effects of our policies and management decisions on water resources at regional and national scales.

The value of NARS data increases with each survey cycle; each additional survey provides new insight into our aquatic resources and future challenges that need to be addressed. Every additional cycle of data strengthens our ability to detect change in our aquatic ecosystems; comparing one survey year to others allows us to understand the proportion and trajectory of improved ecosystems over time. Furthermore, combining all survey years for a single resource enhances the spatial resolution at which we can make estimates (e.g., at smaller scales such as states).

## Conclusions

Over the last 15 years, NARS has amassed continental-extent aquatic resource data using a nationally consistent approach. Even though NARS is primarily an applied monitoring program, it is also a remarkable resource to the scientific community. NARS provides data that help us answer fundamental questions such as whether we are collectively meeting the objectives of the CWA. The data also spur novel insights about our aquatic ecosystems on broad spatial and temporal extents, supporting critical ecological research that advances our understanding of how aquatic systems function and how they may respond to future threats, such as increasing anthropogenic pressures, invasive non-native species, and the climate perturbances (e.g., Chen & Olden, 2020; dos Santos et al., 2024; Feio et al., 2021; Moi et al., 2023, 2024). The portfolio of research projects that use NARS data are testament to the importance of statistically representative, field-based, methodologically consistent, long-term, surveys for detecting patterns at large spatial and temporal extents.

There is great potential in the NARS Program’s future. NARS can integrate emerging technologies like remote sensing and molecular techniques (e.g., environmental DNA (eDNA)) on a national scale, which would help advance the methodologies and protocols for these tools while also collecting cutting-edge data. Because NARS is the only program that collects data across multiple aquatic ecosystems at the national extent, it is well suited to address issues of national importance regarding our aquatic resources. However, like all monitoring programs, logistical, personnel, and funding, constraints remain the largest challenges NARS faces.

To ensure sustenance and continuity, national-scale monitoring programs such as NARS require collaborations across government agencies, states, Tribes, colleges, universities, NGOs, and other partners. NARS is *the* only national extent and consistently applied program to answer questions, with known confidence, about status, trends, extent, risks, and actions – not only about the condition of our aquatic ecosystems, but also, for example, about emerging contaminants of concern in our waterways, climate effects, and other community-based issues. NARS has nearly two decades of baseline data to which new data can be compared. With continued support and recognition of the importance of this premiere national program, the utility of NARS will only grow with time.

## Supplementary Information

Below is the link to the electronic supplementary material.

Supplementary file1 (DOCX 20 KB)

## Data Availability

Data sets collected as part of the National Aquatic Resource Surveys (NARS) Program and reported in this article are cited and available at the following URLs: —United States Environmental Protection Agency. 2022. National Aquatic Resource Surveys. National Lakes Assessment 2017 (“NLA 2017 Condition Estimates (csv)” data and “NLA 2017 condition estimate metadata.txt (txt)” metadata files). Available from U.S. EPA web page: [https://www.epa.gov/national-aquatic-resource-surveys/data-national-aquatic-resource-surveys](https:/www.epa.gov/national-aquatic-resource-surveys/data-national-aquatic-resource-surveys).— United States Environmental Protection Agency. 2023. National Aquatic Resource Surveys. National Rivers and Streams Assessment 2018–19 (“NRSA 1819 Data for Population Estimates (csv)” data and “NRSA 1819 Data for Population Estimates metadata (txt)” metadata files). Available from U.S. EPA web page: [https://www.epa.gov/national-aquatic-resource-surveys/data-national-aquatic-resource-surveys](https:/www.epa.gov/national-aquatic-resource-surveys/data-national-aquatic-resource-surveys).—United States Environmental Protection Agency. 2024a. National Aquatic Resource Surveys. National Coastal Condition Assessment (including the National Great Lakes Assessment) 2020 (“NCCA 2020 Condition Estimates (csv)” data and “NCCA 2020 condition estimate metadata.txt (txt)” metadata files). Available from U.S. EPA web page: [https://www.epa.gov/national-aquatic-resource-surveys/data-national-aquatic-resource-surveys](https:/www.epa.gov/national-aquatic-resource-surveys/data-national-aquatic-resource-surveys).—United States Environmental Protection Agency. 2024b. National Aquatic Resource Surveys. National Wetland Condition Assessment 2021 (“NWCA 2021 Condition Estimates—Data (csv)” data and “NWCA 2021 Condition Estimates—Metadata (txt)” metadata files). Available from U.S. EPA web page: [https://www.epa.gov/national-aquatic-resource-surveys/data-national-aquatic-resource-surveys](https:/www.epa.gov/national-aquatic-resource-surveys/data-national-aquatic-resource-surveys).

## Declarations

All authors have read, understood, and have complied as applicable with the statement on “Ethical responsibilities of Authors” as found in the Instructions for Authors.
