# Supplementary material for: National Aquatic Resource Surveys (NARS): the foundation for long-term aquatic monitoring data across the United States
Source: Environ Monit Assess. 2025 Nov 3;197(12):1291. doi: 10.1007/s10661-025-14629-8 (PMC12583327; doi:10.1007/s10661-025-14629-8)
Supplement: Supplementary file 1 — Supplementary file1 (DOCX 20 KB) [file 10661_2025_14629_MOESM1_ESM.docx]

**Supplementary Table 1** National Aquatic Resource Survey (NARS) webpage descriptions, URLs, and potential uses.

| Webpage Description | URL | Potential Uses |
| --- | --- | --- |
| Field and Laboratory Operation Manuals, Site Evaluation Guidelines, and Design Documents | <https://www.epa.gov/national-aquatic-resource-surveys/manuals-used-national-aquatic-resource-surveys> | Understanding (1) protocols used in the field and laboratory to collect and analyze data, (2) the survey designs including site selection, and (3) evaluation guidelines used to verify targeted sites |
| Map of Past Sampling Locations | <https://www.epa.gov/national-aquatic-resource-surveys/map-national-aquatic-resource-surveys-sampling-locations> | Searching for sampled sites within specific locations, obtaining quick information (e.g., site identifiers such as UID, UNIQUE_ID, and SITE_ID) about a specific site that may be used to query data in the published datasets |
| Published Datasets | <https://www.epa.gov/national-aquatic-resource-surveys/data-national-aquatic-resource-surveys> | Conducting research based on specific data collected in the field or analyzed in laboratories from samples |
| Frequently Asked Questions about the NARS Data, including Technical Support Documents | <https://www.epa.gov/national-aquatic-resource-surveys/frequent-questions-about-data-national-aquatic-resource-surveys#technicaldocuments> | Finding answers about the published datasets, including Technical Support Documents for each survey, which describe the analysis methods used to produce condition thresholds and final condition and trend results |
| Reports and Dashboards, Factsheets and Infographics, Storymaps, Webcasts, and Videos | <https://www.epa.gov/national-aquatic-resource-surveys/outreach-materials-national-aquatic-resource-surveys#reports> | Learning about final condition and trend results from the surveys, published as reports and interactive dashboards, and other associated information about the surveys (e.g., from factsheets, infographics, storymaps, webcasts, and videos) in a way that is accessible to multiple audiences, from the public to scientists |
| Journal Articles relating to NARS or using NARS data | <https://www.epa.gov/national-aquatic-resource-surveys/journal-articles-applying-national-aquatic-resource-survey-data> | Conducting literature searches for NARS specific and NARS related peer-reviewed articles, organized by aquatic ecosystem type and chronologically |
| NARS Tools, Training Videos, Links to StreamCat and LakeCat | <https://www.epa.gov/national-aquatic-resource-surveys/tools-related-national-aquatic-resource-surveys> | Using user-friendly R Shiny apps to apply NARS survey designs, thresholds, and other methodology to your own datasets; watching NARS field training videos; accessing supplemental datasets that provide local catchment and watershed metrics for streams (StreamCat) and lakes (LakeCat) |
